# Bionomics and ecology of *Anopheles merus* along the East and Southern Africa coast

**DOI:** 10.1186/s13071-021-04582-z

**Published:** 2021-01-28

**Authors:** Brian Bartilol, Irene Omedo, Charles Mbogo, Joseph Mwangangi, Martin K. Rono

**Affiliations:** 1 Kenya Medical Research Institute, Centre for Geographic Medicine Research-Coast, Kilifi, Kenya; 2 Pwani University Bioscience Research Centre (PUBReC), Pwani University, Kilifi, Kenya; 3 Big Data Institute, University of Oxford, Oxford, UK; 4 Kenya Medical Research Institute, Centre for Vector Disease Control, Kwale, Kenya

**Keywords:** *Anopheles merus*, *Plasmodium falciparum*, Indoor residual spraying, Insecticide resistance

## Abstract

Malaria transmission persists despite the scale-up of interventions such as long-lasting insecticide-treated nets (LLINs) and indoor residual spraying (IRS). Understanding the entomological drivers of transmission is key for the design of effective and sustainable tools to address the challenge. Recent research findings indicate a shift in vector populations from the notorious *Anopheles gambiae* (*s.s.*) as a dominant vector to other species as one of the factors contributing to the persistence of malaria transmission. However, there are gaps in the literature regarding the minor vector species which are increasingly taking a lead role in malaria transmission. Currently, minor malaria vectors have behavioural plasticity, which allows their evasion of vector control tools currently in use. To address this, we have reviewed the role of *Anopheles merus*, a saltwater mosquito species that is becoming an important vector of malaria transmission along the East and Southern African coast. We performed a literature review from PubMed and Google Scholar and reviewed over 50 publications relating to *An. merus*'s bionomics, taxonomy, spatial-temporal distribution and role in malaria transmission. We found that *An. merus* is an important vector of malaria and that it contributes to residual malaria transmission because of its exophilic tendencies, insecticide resistance and densities that peak during the dry seasons as the freshwater mosquitoes decline. Spatial and temporal studies have also shown that this species has increased its geographical range, densities and vectorial capacity over time. In this review, we highlight the resting behaviour and breeding habitats of this mosquito, which could be targeted for surveillance studies and control interventions. 
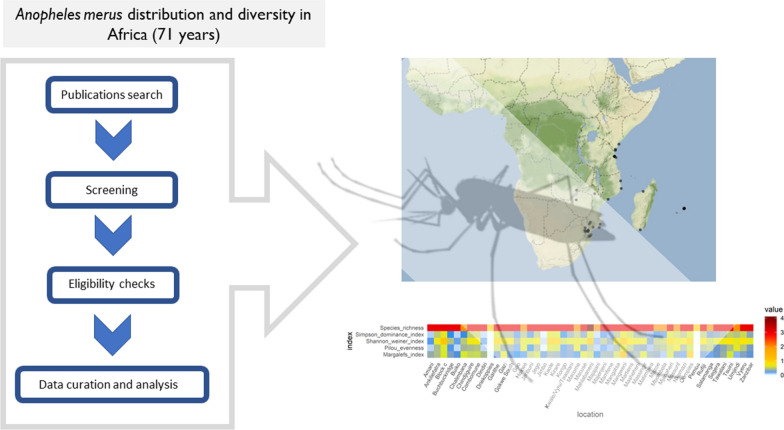

## Background

Malaria continues to be a significant cause of morbidity and mortality in Africa, especially in sub-Saharan Africa [1]. In 2018, over 400,000 malaria deaths were reported globally of which 67% were reported in children under 5 years [1]. Global malaria control has historically been undertaken to prevent new infections and to ensure that malaria transmission is curtailed. Malaria control and eradication efforts have gone through different phases from which several lessons have been learnt. The initial campaigns in the early twentieth century were country-specific and were not well funded. For example, in 1939–1940, Fred Soper of the Rockefeller Foundation led vigorous larval source management (LSM) activities using the dust larvicide, Paris green (copper(II) acetoarsenite), leading to the elimination of *Anopheles gambiae* from Brazil [2]. LSM was effective but tedious in its implementation, which necessitated the discovery and deployment of other measures to control mosquito vectors and to diagnose and treat malaria infections [3, 4]. During World War II (WWII), much of the previously achieved gains in vector control were lost because of the shift of investments to the war, destruction of infrastructure and the displacement of people. After the end of WWII, world bodies such as the United Nations (UN) and the World Health Organisation (WHO) were created to pursue world security issues and coordinate health-related issues respectively. To ensure control and eradication of malaria post-WWII, WHO, during the 8th World Health Assembly in 1954 [5], resolved to have a consolidated effort to eradicate malaria globally, which was implemented between 1955 and 1972 [6–8]. Several attempts to undertake indoor residual spraying (IRS) were made especially between 1955 and 1969, for instance, the Pare-Taveta Malaria Control Scheme on the Kenyan and Tanzanian border, which was carried out by the East Africa Institute of Malaria and Vector Borne Diseases (EA-MVBD) and the WHO [7]. This large-scale trial undertook residual spraying (IRS) with Dieldrin and DDT in the Taveta sub-district of Kenya and the Pare district of Tanzania. During this expansive malaria control programme, entomological and parasitological surveillance systems were deployed to monitor the decline in malaria cases and malaria transmission. The programme's impact analysis showed that human mortality among all age groups was halved during the spraying campaign. The entomological surveys showed that *An. funestus* was eliminated by the IRS programme while the *An. gambiae* population was reduced sevenfold, and sporozoites were undetectable in the infected population [9]. After the collapse of the first Global Malaria Eradication Program, malaria control efforts were undertaken through interventions against the disease causing the malaria parasite, such as the use of chloroquine for case management [10]. However, treatment failures were reported due to the development of chloroquine resistance [11]. The current global malaria control initiative was re-established in the late 1990s through consulted leadership of the WHO, with the establishment of consolidated efforts such as Roll Back Malaria (RBM). These WHO-mediated efforts changed the malaria control paradigm and ensured malaria control was implemented through a programmatic approach. During this phase, several multi-faceted strategies were implemented targeting enhancing partnerships, government/political support such as the Abuja Declaration by the continent’s heads of state [12], control of vectors (scale-up of ITN/LLINs, IRS), case detection, case management, major campaigns in advocacy communication and social mobilization for behaviour change [13, 14]. At the national level, malaria control efforts were managed through National Malaria Control Programmes (NMCPs), which worked closely with WHO. These efforts have led to a significant decline in malaria morbidity and mortality, especially between 2000 and 2015 [15]. Although malaria-endemic countries are currently faced with an upsurge in malaria as a result of residual transmission [1, 16], there is a renewed effort to contain malaria, especially towards pre-elimination and elimination phases as outlined by the WHO Global Technical Strategy for Malaria 2016–2030 [17].

Over the decades, vector control has become a major pillar in the fight against malaria and has mainly been done through scaling up the use of LLINs and IRS. The indoor-based chemical control interventions resulted in a tremendous decline in malaria prevalence and incidence especially between 2000 and 2015 [15]. However, malaria vectors have responded via behavioural modifications, including changing their feeding and resting patterns [7, 8, 18, 19]. Additionally, changes in species composition, which has seen previously minor malaria species become more dominant (species replacement) [20–22], as well as the development and spread of insecticide resistance have all contributed to an increase in malaria incidence post 2015 [23–27].

Traditionally, the dominant vectors of malaria have been *An. gambiae* (*s.s.*) and *Anopheles funestus*, which preferentially feed on humans and reside indoors. Whilst most known interventions focusing on indoor feeding and resting mosquitoes remain effective against the two mosquito species, changes in vector composition and behaviour have seen the decline of *An. gambiae* (*s.s.*) and the emergence of new vectors.

Some members of the *An. gambiae* complex (*An. merus*) and other species (*An. coustani, An. pretoriensis and An. moucheti)* that were initially considered as minor malaria vectors have recently been reported to play a leading role in malaria transmission [21, 22, 28, 29]. Behaviour modification to favour outdoor feeding and resting and reliance on blood-meal sources from humans to alternate vertebrates [19, 22] have allowed the new vectors to avoid LLINs that largely target indoor feeding and resting mosquitoes. However, these minor species have been given little attention in malaria research, resulting in a paucity of information about their biology, behaviour and role in disease transmission. As these species gain dominance, this knowledge will be crucial in the design and application of control interventions. This review has focused on the identification methods, bionomics, spatial and temporal distribution and the role in disease transmission of *Anopheles merus*, which is increasingly becoming an important malaria vector in the East and Southern coastal regions of Africa.

## Methods

### Search strategy and selection criteria

The electronic databases PubMed and Google Scholar were searched for articles documenting *An. merus* using the search phrases “Anopheles merus” and “saltwater Anopheles gambiae”. All the documents were screened and assessed to determine whether they had any data on vector densities, breeding sites, vectorial capacity and the coordinates of mosquito collection sites.

### Data analysis

Data were extracted from the articles and stored in a Microsoft Excel file. The coordinate data were converted into a similar format (Decimal Degrees) using the Polar Geospatial Center (PGC) Coordinate Converter [30]. Statistical analysis and data visualisation were carried out using the R software, version 3.6.3 [31]. Comparisons between the different mosquito counts were done using the Kruskal-Wallis test [32].

The mosquito population structure in each of the sites was analysed using the following ecological parameters: population abundance (total number of mosquitoes per site), species richness (measure of the number of species per site), species evenness (measure of how homogeneous a community is in terms of abundance of all its species) and species diversity (Shannon-Weiner index, Simpson dominance index and Margalef’s index).

To measure species evenness, the Pielou index was used, which compares the actual diversity values using Shannon-Wiener index to the maximum diversity value. The Pielou index value ranges from 0 to 1, the more the variation in abundance between the different taxa within the community the lower the *J* value and vice versa [33].

$$J=\frac{H^{\prime}}{H_{max}}$$

where *H’* is the number derived from the Shannon-Wiener diversity index and *H*_*max*_ is the maximum possible value of *H’*, which is derived from the equation:

$$H_{max} = \mathop \sum \limits_{i = 1}^{s} \frac{1}{S}ln\frac{1}{S} = lnS$$

The Margalef’s index measures only gross species richness [34]. The equation is as follows

$$D_{Mg} = \frac{{\left( {S - 1} \right)}}{ln\left( N \right)}$$

where *S* is the number of species and *N* is the number of mosquitoes in the collection sites.

The Shannon-Weiner index characterises the diversity of a community by considering the abundance and evenness of species present. The index increases as both the richness and evenness of the community increases. The values range from 0 to 5 [35]. The equation:

$$H^{\prime} = \mathop \sum \limits_{i = 1}^{S} p_{i} \left( {lnp_{i} } \right)$$

where *p*_*i*_ is the proportion of individuals belonging to the *i*th species and *S* is the number of species.

The Simpson diversity index measures the degree of dominance of an individual mosquito species using the following equation [36]:

$$\lambda = \mathop \sum \limits_{i = 1}^{S} p_{i}^{2}$$

where *p*_*i*_ is the proportion of individuals belonging to the ith species and S is the total number of species.

## Results

**Fig. 1:**
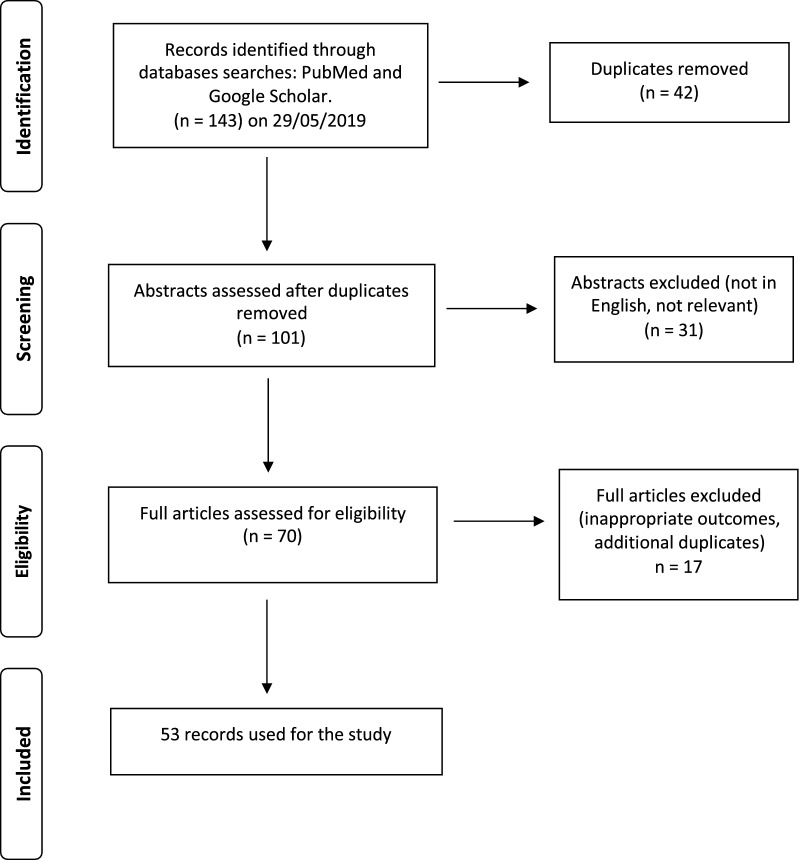
Flow chart describing the database search and selection of the articles used in the literature review

A total of 143 records were found during the initial search with 42 of these being duplicates which were dropped. The abstracts of the remaining records were screened and out of these 70 were retained and the rest removed since inappropriate outcomes were assessed or not in English. The full articles of the remaining documents were screened and out of these we remained with 53 articles which were included in the study, and the data were extracted and are available in Additional file 1. The results of the search strategy are provided in a PRISMA flow chart (Fig. 1).

### Identification of *An. merus*

Initially *An. merus* was distinguished from other *An. gambiae* (*s.l.*) sibling species using a salinity physiological method whereby the hatched larvae were transferred into 75% seawater (23.8 g NaCl/l). Those that died within 2 h were classified as freshwater forms while those that survived for about 6 h were classified as saltwater forms [37]. Later, emphasis was put on taxonomic characteristics with the number of antennal sensilla, palp ratio and shape of the egg being the most reliable way of differentiating saltwater and freshwater *An. gambiae*. However, these characteristics are concordant with both saltwater species, *An. merus* and *An. melas*, except for the ornamentation of tarsi in which *An. merus* is richer in white scales and in the larval chaetotaxy [38]. The banding patterns of the polyene chromosomes in the ovarian nurse cells of half gravid females have also been used to distinguish the members of the *An. gambiae* complex [39] although this could not distinguish male mosquitos. Mahon et al. [40] improved on this limitation using allozymes to identify adults of *An. gambiae* complex regardless of sex and gonotrophic cycle. Hybridization reactions using DNA probes that were species-specific or revealed species-specific restriction enzyme fragments were used [41–43]. In 1993, Scott et al. developed a ribosomal DNA- polymerase chain reaction method that identified the members of *An. gambiae* complex using species-specific nucleotide sequences in the ribosomal intergenic spacers [44]. The advantage of the procedure is that it is sensitive, since most mosquito species have 500 or more copies of ribosomal DNA units and it also utilises both extracted and non-extracted specimens as templates for PCR with a sensitivity of > 95% and > 85% respectively. Recently, advances in proteomics have led to new methods based on whole cells: matrix-assisted laser desorption/ionisation time-of-flight mass spectrometry (MALDI-TOF MS). This approach relies on shrinkage discriminant analysis procedure for differentiating Anopheline species and resolving colony-specific patterns [45]. Near-infrared spectroscopy has also been used for speciation and ageing of *An. gambiae* (*s.s.*) and *An. arabiensis* [46]. Loop-mediated isothermal amplification (LAMP), which is performed in isothermal conditions, has been used to identify *An. gambiae* (*s.s.*) and *An. arabiensis*. The strength of the LAMP technique is attributed to the high sensitivity and requires simple equipment, making it suitable for field applications with limited access to the more expensive thermocyclers [47].

### Species distribution and diversity

**Fig. 2:**
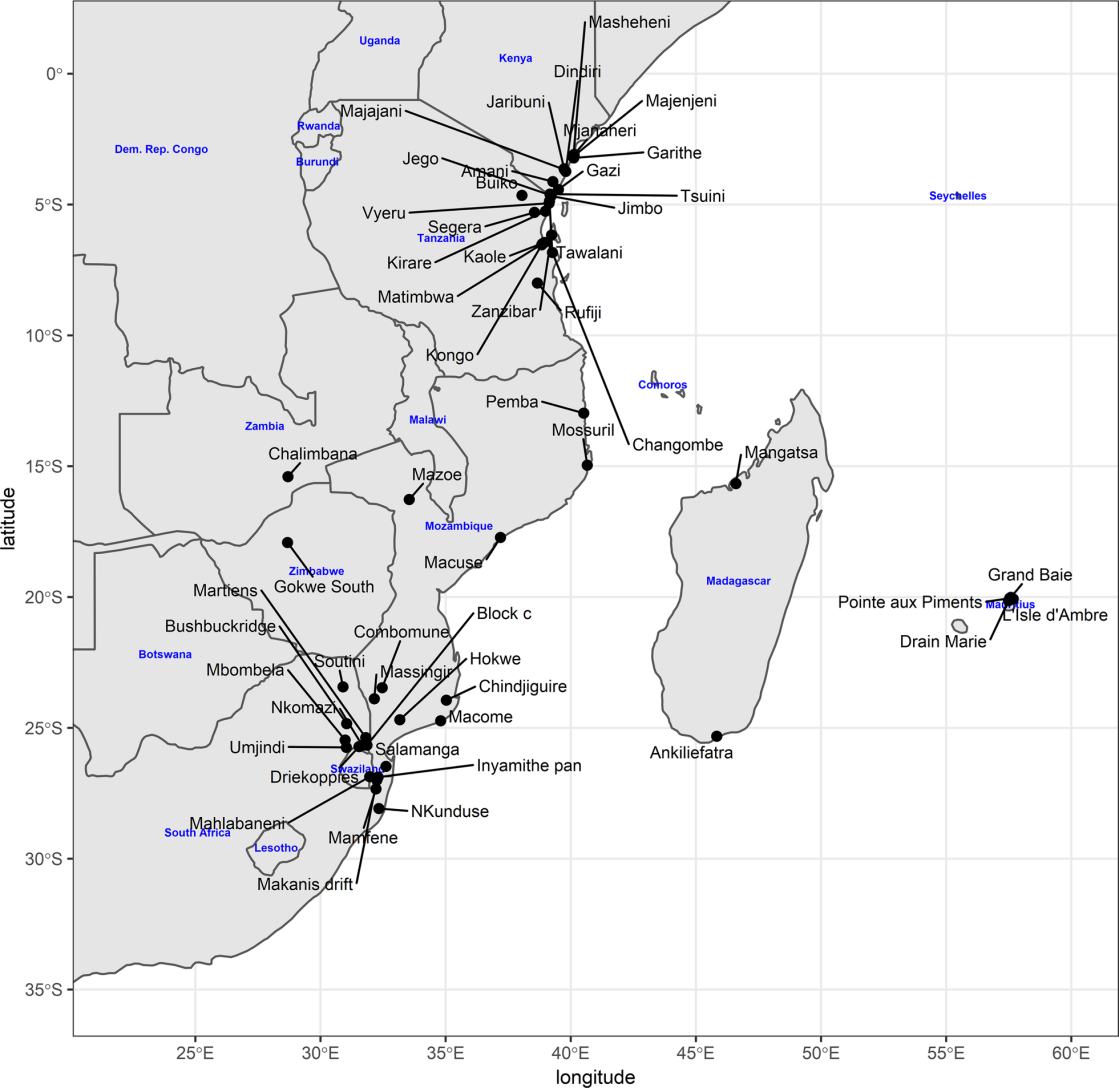
Map depicting sites where *An. merus* have been identified from mosquito collections along the East and Southern African Coast

**Fig. 3:**
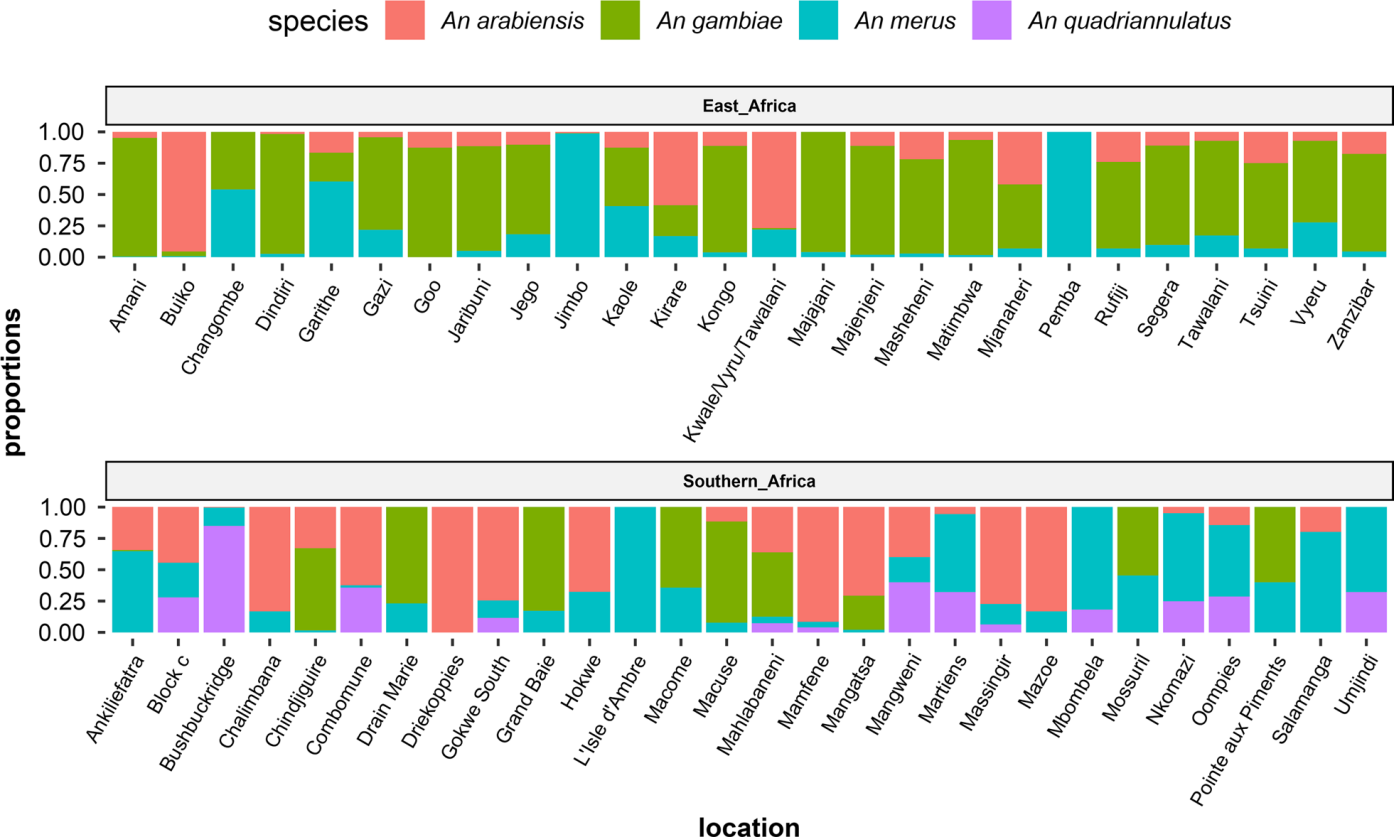
Proportions of *An. gambiae* complex members: *An. gambiae* (*s.s.*), * An. merus, An. quadriannulatus* and *An. arabiensis* in the different collection sites along the East and Southern African Coast. However, in Changombe, Drain Marie, Grand Baie, L’Isle’d’Ambre, Pemba and Pointe aux Piments, *An. gambie* (*s.l.*) were identified using a salt tolerance test and classified as saltwater *An. gambiae* represented as *An. merus* in the figure or non-saltwater *An. gambiae* (*An. arabiensis, An. quadriannulatus, An. gambiae*), which are represented as *An. gambiae*

The sites from which *An. merus* were obtained are shown in Fig. 2 and Additional file 1. Furthermore, the proportion of these species in the different sites is illustrated in Fig. 3. Comparisons on the number of species in each site show that the Mahlabaneni region has the most species (*An. gambiae, An. merus, An. quadriannulatus* and *An. arabiensis*) while Driekoppies has only one species (*An. merus)*.

**Fig. 4:**
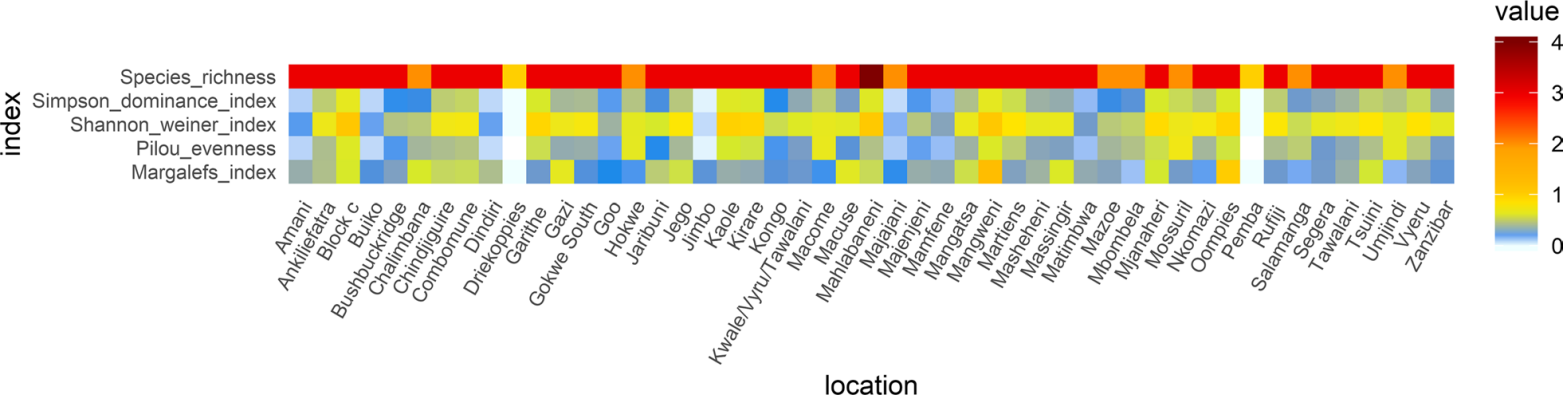
Species distribution and diversity indices of *An. gambiae* (*s.l.*) collections in sites along the East and Southern African Coast. Analysis was only done for sites where mosquitoes were identified through PCR or cytotaxonomy

Tests on species richness using Margalef's index show that the richest areas were Mangweni and Oompies D_*mg*_ *=* 1.24 and 1.03 respectively with the least rich areas being Umjindi, Mbombela and Driekoppies (0–0.16) (Fig. 4)*.* Analysis of species diversity: Shannon-Wiener index (*H'*) and Simpson's index (*λ*) of the different sites were performed. The most diverse areas were Mangweni, Kaole and Mahlabaneni where the range was *H'* (0.98–1.06) and (*λ*) (0.59–0.65) (Fig. 4). The least diverse areas were Majajani, Jimbo and Driekoppies. Tests on the homogeneity/evenness of the species in these sites using the Pielou evenness (*J*) equation show that the most homogeneous sites were Mossuril (0.72), Macome (0.660, Hokwe (0.63) and Umjindi (0.63) with the least even areas being Dindiri, Jimbo and Driekoppies (Fig. 4).

### Resting behaviour and biting cycle

Studies in Dar-es-salaam and Pemba have shown that a significant numbers of *An. merus* leave the house at dawn after feeding the night before [8, 37]. In Jimbo, they came to a similar conclusion as a number of blood-fed *An. merus* were found outdoors [48]. In Pemba, a large proportion of the mosquitoes collected were resting outdoors under mangrove, mango trees, fallen leaves and coralline rocks and in crab holes [8], whereas in Garithe both indoor and outdoor resting was observed [28]. These similarities in the resting tendencies between indoor and outdoor mosquitoes have been attributed to an intrinsic rhythm or homogeneity in the indoor and outdoor populations [48].

Regarding the biting cycle, two studies came to similar conclusions in South Africa and Kenya. In NKunduse, South Africa, the lowest catches occurred between 18:00 h and 22:00 h, increased significantly between 22:00 h and 02:00 h and then dropped afterwards [49]. In Jimbo village of Kenya, both indoor and outdoor biting activity started to rise at 18:00 h attaining the peak between 24:00 h and 01:00 h and declined gradually to 06:00 h [48]. The normal cycle is however dynamic and is influenced by environmental factors such as wind and temperature [49].

### The role of *An. merus* in malaria transmission

Various studies have documented the role of *An. merus* as a vector of malaria. In Boane, Mozambique, sporozoite rates of 4.2% for *An. merus*, 9.6% for An arabiensis and 4.3% for *An. funestus* were observed . Additionally, comparisons of oocyst rates showed that there was no significant difference among the three mosquito vector species [50]. In Tanzania, sporozoite rates for *An. merus* have been reported to range between 1.5 and 11.6 [37]. In Ankiliefatra, Madagascar, out of 275 *An. gambiae* (*s.l.*) analysed for circumsporozoite antigen, only two female *An. merus* collected indoors and outdoors were positive [51]. Sporozoite rates ranging between 1 and 29.3% have been reported in Kenya with the highest rates observed in Garithe village [29].

The increase in geographical range and population densities of *An. merus* has potential for this species to play a significant role in malaria transmission [28, 29, 52, 53]. For example, during the dry seasons the densities of *An. merus* peak as the freshwater species diminish; therefore, *An. merus* may potentially sustain malaria transmission during the dry season [20, 54].

### *An. merus* as a vector for lymphatic filariasis

Lymphatic filariasis is a neglected tropical disease which causes painful and disfiguring visible manifestations and permanent disability [55]. By 2018, the disease was a threat to 12% of the global population [56]. The first observation of the transmission of filariasis by *An. merus* was in Tanzania in 1948 when filarial larvae were detected in 22% of the saltwater forms of *An. gambiae* compared to 6% in the freshwater forms [37]. In a study carried out in Vyeru village on the Tanzanian coast, comparisons between *An. merus* and *An. gambiae* collected both outdoors and indoors showed that *An. merus* was a more efficient vector of bancroftian filariasis [57]. In Jimbo and Jego villages of Kenya, findings differed from those of Vyeru where *An. gambiae* was ten times more efficient than *An. merus* [58]. Additional studies on the importance of *An. merus* as vectors of transmission of diseases such as lymphatic filariasis are required, given the evidence of their increasing dominance.

### Insecticide resistance

Only one study could be found describing the response of *An. merus* to pyrethroids (deltamethrin), carbamates (bendiocarb) and organochlorines (DDT) as well as this one in South Africa [54]. The species was fully susceptible to deltamethrin and bendiocarb, with 97% mortality on DDT, suggesting possible resistance developing to this insecticide. Since the species is mostly exophilic and therefore less likely to come into contact with the insecticides used for IRS and ITNs, it is suggested that the possible DDT resistance could be driven by selective pressure that may have occurred at the larval stage because of environmental contamination [54].

### Larval ecology

The presence of *An. merus* larvae in a particular habitat is determined by suitable physiochemical and other environmental factors with the most significant determinants being temperature, salinity, algae, conductivity and canopy coverage [59]. Warmer temperatures have been found to accelerate the development of the larvae and proliferation of microorganisms that provide nutrients for the larvae as well as speeding up the decomposition of leaves, debris and algae, which also act as a food source [60–62]. For example, the presence of algae in pools has been associated with the presence of *An. merus* larvae [59]. It has also been shown that algal biomass and abundances and microeukaryote community structure are influenced by larval grazing [63].

Rain has been shown to influence salinity, whereby during the onset of rains, salts deposited at the bottom of semi-permanent breeding sites become dissolved creating favourable breeding salinities; however, long rains dilute the brackish water consequently reducing *An. merus* densities. As the rains disappear, the semi-permanent breeding sites start evaporating, increasing salinity achieving optimum conditions sustaining increased *An. merus* densities. However, high salinity levels are associated with reduced mosquito densities [64]. In Dar-es-salaam, the saltwater forms of *An. gambiae* were shown to be dominant during the dry season (September–August) while the freshwater forms were dominant during the wet season (May–June) [8].

*Anopheles merus* has long been known to breed in brackish water in the Eastern and Southern Coast of Africa and further inland [8, 37, 59, 62, 65, 66]. Larvae have been identified from breeding in a wide range of salinities(percent seawater): between 60 and 186% in Tanzania [8, 37, 67, 68], 46% in Jubaland [69], 60% in Swaziland [65], 18–205% in Mauritius [70–72], 30–96% in Kenya [64, 73] and 1.8–123% in South Africa [65, 74]. Larval survival in saline environments relies on regulation of haemolymph osmolarity through the intake absorption and excretion of ions in the rectum. Comparisons between fresh and saline water reared larvae showed that there was a dramatic shift in the rectal Na+/K+-ATPase protein localization [75]. It has also been shown that phenotypic tolerance of *An. merus* to saltwater is highly dependent on the timing of larval exposure to salinity, specifically within the first 24 h [76].

Various chemicals other than sodium chloride found in seawater have been thought to affect the development of *An. merus*. In Jimbo, Kenya, Mosha and colleagues [64] showed the importance of these salts (sodium chloride, potassium chloride, magnesium chloride, sodium sulphate, magnesium sulphate, sodium carbonate and potassium carbonate) in the survival of the larvae by comparing saltwater and seawater where the median lethal dose was 102.5% and 135.0% salinity respectively, suggesting that some chemicals in seawater might enable the tolerance of higher concentrations of sodium chloride. Earlier studies had also indicated that tolerance in some forms of *An. maculipennis* was improved by addition of minute amounts of calcium (in form of carbonates, chlorides or sulphates) [77]. For *An. melas* it was identified that magnesium sulphate was attractive to ovipositing females [78]. Toxicity tests also showed that sodium sulphate and magnesium sulphate were well tolerated and supported eclosion with less mortality than sodium chloride of the same concentration [64]. Recently, Jeanrenaud et al. showed that larval exposure to organic fertiliser led to an increase in the adult lifespan [79].

### Habitats along the East and Southern African coast

There have been various accounts of *An. merus* along the Kenyan coast. In 1983, the species was identified in Jimbo village, Kwale district, both at the shoreline, which is made up of mangrove vegetation, and further inland in large semi-permanent brackish ponds [48]. In 2003, Mbogo and colleagues [29] reported the species in three coastal districts: Malindi, Kilifi and Kwale. In Kilifi, it was reported in Dindiri, Jaribuni and Majajani where the Jaribuni River and swamps along it turn saline during the dry season, in Malindi at Garithe, Manjenjeni, Masheheni and Mjanaheri while in Kwale at Amani, Gazi and Tsuini village. In Garithe village of Kilifi county, the species was identified in manmade pools, road drains, ponds swamps and hoofprints [59]. The localization of the species to the coastal region was suggested to be due to strict adaptation to saltwater breeding or competitive exclusion further inland by other members of the *An. gambiae* complex [58]. Wind-assisted dispersal of *An. merus* further inland was ruled out because of dense vegetation but with sparse vegetation, a mark and release experiment showed that the species can travel between 4.5 and 7 km [80].

In Changombe village in Dar-es-salaam, Tanzania, it was shown that the species breeding sites periodically occurred in swampy patches along the upper tidal limit that was only reached by the highest spring tides, around mangrove bushes and more than half a kilometre inland in shallow, brackish water ponds. The breeding season was usually in August, September and October [37]. The species was also identified in Kaole village where they concluded that *An. merus* at the coast survived longer than those further inland [81]. In Zanzibar, Pemba Island, the rugged coastline facilitates entry of seawater to the island during spring tides leaving saltwater pools that provide breeding sites for *An. merus* [8].

In Swaziland, the species has been found in pools about 90 km from the coastline and more than 121 km up the Usutu River containing about 63% saltwater [65]. Breeding sites in South Africa have been identified in saline pools at the edge of Inyamithe Pan, Northern Natal [65], and in cattle hoofprints formed along a hot mineral spring in Soutini [82].

In Madagascar, the species was first identified in a salt swamp near Toliara [83] later on at Mangatsa and Ankilifietra [51]. The species was always found ≥ 20 km from the coastline and in areas without mangroves showing that it was not dependent on them; there was no *A. merus* in Kimony, which is covered by a large mangrove forest [51].

In Mauritius, breeding sites were crab holes, former salt pans and further inland in large pools away from the shore. The sites were Baines des Dames, Drain Marie, Pointe aux Piments and L’Isle d’Ambre. The absence of larvae in the breeding sites was attributed to flooding during spring tides and drying up of the pools during low tide [72].

## Conclusion

*An. merus* has been given little attention and very few studies have been done. The paucity of data on *An. merus* and its transmission dynamics needs to be bridged since the species is becoming an important vector of malaria and possibly lymphatic filariasis. It is also displaying both phenotypic and behavioural resistance to insecticides. This therefore calls for the deployment of innovative control strategies such as mass drug administration (endectocides such as ivermectin), larviciding, transgenic mosquitoes, zooprophylaxis, spatial repellents, attractive toxic sugar baits, “eave tubes” and targeting mosquito swarms.

## Supplementary Information

**Additional file 1.** Mosquito collection sites, coordinates, date of collection, mosquito counts, indoor or outdoor collection, mosquito stage and the relevant references.

## Data Availability

Data and analysis files are available in Harvard Dataverse at https://doi.org/10.7910/DVN/1QOXQR.

## Abbreviations

KEMRI: Kenya Medical Research Institute
WHO: World Health Organisation
IRS: Indoor Residual Spray
DDT: Dichloro-diphenyl-trichloroethane
ITN: Insecticide-treated bed nets
PRISMA: Preferred reporting items for systematic reviews and meta-analyses
