# Supplementary material for: Bionomics and ecology of Anopheles merus along the East and Southern Africa coast
Source: Parasit Vectors. 2021 Jan 28;14:84. doi: 10.1186/s13071-021-04582-z (PMC7842043; doi:10.1186/s13071-021-04582-z)
Supplement: Supplementary file 1 — Additional file 1. Mosquito collection sites, coordinates, date of collection, mosquito counts, indoor or outdoor collection, mosquito stage and the relevant references. [file 13071_2021_4582_MOESM1_ESM.docx]

| country | district | location | latitude | longitude | date of collection | An me | An ar | An ga | An qu | indoor/outdoor | stage | article |
| --- | --- | --- | --- | --- | --- | --- | --- | --- | --- | --- | --- | --- |
| Kenya | Kwale | Amani | -4.12353 | 39.28475 | June 1997 to May 1998 | 1 | 10 | 196 | 0 | indoor | adults | Mbogo et al., 2003 |
| Madagascar | Tsihombe | Ankiliefatra | -25.3204 | 45.83333 | March 2003 | 14 | 2 | 0 | 0 | indoor | adults | Leong et al., 2003 |
| Madagascar | Tsihombe | Ankiliefatra | -25.3204 | 45.83333 | March 2003 | 6 | 1 | 0 | 0 | outdoor | adults | Leong et al., 2003 |
| Madagascar | Tsihombe | Ankiliefatra | -25.3204 | 45.83333 | March 2003 | 4 | 0 | 0 | 0 | indoor | adults | Leong et al., 2003 |
| Madagascar | Tsihombe | Ankiliefatra | -25.3204 | 45.83333 | March 2003 | 11 | 12 | 0 | 0 |  | aquatic stages | Leong et al., 2003 |
| Madagascar | Tsihombe | Ankiliefatra | -25.3204 | 45.83333 | March 2003 | 8 | 2 | 1 | 0 |  | adults | Leong et al., 2003 |
| Madagascar | Tsihombe | Ankiliefatra | -25.3204 | 45.83333 | February to September 1996 | 10 | 11 | 0 | 0 | indoor | adults | Leong et al., 2003 |
| Madagascar | Tsihombe | Ankiliefatra | -25.3204 | 45.83333 | February to September 1996 | 9 | 7 | 0 | 0 | outdoor | adults | Leong et al., 2003 |
| Madagascar | Tsihombe | Ankiliefatra | -25.3204 | 45.83333 | February to September 1996 | 5 | 0 | 0 | 0 | indoor | adults | Leong et al., 2003 |
| Madagascar | Tsihombe | Ankiliefatra | -25.3204 | 45.83333 | February to September 1996 | 5 | 3 | 0 | 0 |  | adults | Leong et al., 2003 |
| South_Africa | Tonga | Block c | -25.6606 | 31.85806 | August1997 to May1998 | 10 | 16 | 0 | 10 | indoor/outdoor | adults | Govere et al., 2000 |
| South_Africa | Ehlanzeni | Bushbuckridge | -24.8398 | 31.04641 | 2005- 2017 | 56 | 1 | 0 | 324 |  | adult/larvae | Mbokazi et al., 2018 |
| Tanzania | Tanga | Buiko | -4.65 | 38.05 | April 1982 to January 1985 | 7 | 907 | 37 | 0 | indoor | adults | Mnzava et al., 1986 |
| Zambia | Chongwe | Chalimbana | -15.4 | 28.7 | March 1995 | 1 | 5 | 0 | 0 |  | larvae | Kloke et al., 1997 |
| Tanzania | Dar_es_salaam | Changombe | -6.833 | 39.267 | 1947- 1948 | 533 | 0 | 453 | 0 | indoor | adult | Muirhead et al., 1951 |
| Mozambique | Boane | Chindjiguire | -23.9422 | 35.02889 | 02/02/2002 | 1 | 20 | 40 | 0 | indoor | adult | Cuamba et al., 2009 |
| Mozambique | Boane | Combomune | -23.4664 | 32.46528 | 17/07/2001 | 1 | 35 | 0 | 20 | outdoor | larvae | Cuamba et al., 2009 |
| Kenya | Kilifi | Dindiri | -3.73333 | 39.8 | June 1997 to May 1998 | 3 | 2 | 110 | 0 | indoor | adults | Mbogo et al., 2003 |
| Mauritius | Drain Marie | Drain Marie | -20.1431 | 57.49167 | 1963 | 3 | 0 | 10 | 0 | outdoor | adults | Patterson et al., 1963 |
| South_Africa | Shongwe | Driekoppies | -25.7167 | 31.5403 | August1997 to May1998 | 0 | 19 | 0 | 0 | indoor/outdoor | adults | Govere et al., 2000 |
| Kenya | Malindi | Garithe | -3.21919 | 40.11689 | September 2007 to March 2008 | 268 | 28 | 36 | 0 | indoor/outdoor | adults | Kipyab et al., 2013 |
| Kenya | Malindi | Garithe | -3.21919 | 40.11689 | June 1997 to May 1998 | 66 | 63 | 91 | 0 | indoor | adults | Mbogo et al., 2003 |
| Kenya | Kwale | Gazi | -4.42402 | 39.50628 | June 1997 to May 1998 | 5 | 1 | 17 | 0 | indoor | adults | Mbogo et al., 2003 |
| Zimbabwe | Gwave | Gokwe South | -17.9167 | 28.68333 | Feb-06 | 22 | 470 | 0 | 73 | indoor/outdoor | adults/larvae | Munhenga et al., 2008 |
| Zimbabwe | Gwave | Gokwe South | -17.9167 | 28.68333 | Jan-08 | 107 | 209 | 0 | 32 | indoor/outdoor | adults/larvae | Munhenga et al., 2008 |
| Tanzania | Tanga | Goo |  |  | apr-1982 to jan-1985 | 2 | 218 | 1501 | 0 | indoor | adults | Mnzava et al., 1986 |
| Mauritius | Grand Baie | Grand Baie | -20.0131 | 57.58444 | 1963 | 9 | 0 | 43 | 0 | outdoor | adults | Patterson et al., 1963 |
| Mozambique | Boane | Hokwe | -24.6903 | 33.16972 | 11/06/2000 | 23 | 48 | 0 | 0 | outdoor | adult | Cuamba et al., 2009 |
| South_Africa | Northern natal | Inyamithe pan | -26.8833 | 32.3 | 2005- 2019 |  |  |  |  | outdoor | adults | Mbokazi et al., 2018 |
| Kenya | Kilifi | Jaribuni | -3.63333 | 39.73333 | June 1997 to May 1998 | 4 | 9 | 66 | 0 | indoor | adults | Mbogo et al., 2003 |
| Kenya | Kwale | Jego | -4.64376 | 39.18693 | 1980 | 9 | 5 | 35 | 0 | indoor/outdoor | adults | Mosha et al., 1983 |
| Kenya | Kwale | Jimbo | -4.67361 | 39.21278 | 1978 - 1981 | 769 | 7 | 3 | 0 |  | adults | Mosha et al., 1983 |
| Tanzania | Bagamoyo | Kaole | -6.45 | 38.95 | June to SSeptember 1996 | 84 | 26 | 96 | 0 | indoor | adult | Temu et al., 1998 |
| Tanzania | Tanga | Kirare | -5.25592 | 38.98862 | jun-05 to dec-06 | 28 | 62 | 58 | 0 | indoor | adults | Derua et al., 2012 |
| Tanzania | Tanga | Kirare | -5.25592 | 38.98862 | January 2007 to April 2011 | 14 | 83 | 3 | 0 | indoor | adults | Derua et al., 2012 |
| Tanzania | Bagamoyo | Kongo | -6.54 | 38.84 | June to September 1996 | 32 | 95 | 729 | 0 | indoor | adult | Temu et al., 1998 |
| Tanzania |  | Kwale/Vyeru/Tawalani | | | June 2011 | 129 | 449 | 7 | 0 | indoor | adults | Derua et al., 2012 |
| Mauritius | L'Isle d'Ambre | L'Isle d'Ambre | -20.0742 | 57.68028 | 1963 | 8 | 0 | 0 | 0 | outdoor | adults | Patterson et al., 1963 |
| Mozambique | Boane | Macome | -24.7292 | 34.80194 | 16/03/2001 | 21 | 0 | 38 | 0 | indoor | adult | Cuamba et al., 2009 |
| Mozambique | Boane | Macuse | -17.7233 | 37.19056 | 26/07/2000 | 2 | 3 | 21 | 0 | indoor | adult | Cuamba et al., 2009 |
| Swaziland | Vryheid East | Mahlabaneni | -26.8667 | 31.96667 | 01/09/1991 to June 1992 | 22 | 154 | 220 | 31 |  | larvae | Grange et al., 1995 |
| Kenya | Kilifi | Majajani | -3.66667 | 39.75 | June 1997 to May 1998 | 2 | 0 | 48 | 0 | indoor | adults | Mbogo et al., 2003 |
| Kenya | Malindi | Majenjeni | -3.13668 | 40.13439 | June 1997 to May 1998 | 4 | 23 | 179 | 0 | indoor | adults | Mbogo et al., 2003 |
| South_Africa | uMkanyakude | Mamfene | -27.333 | 32.217 | January 2017 to May 2018 | 11 | 228 | 0 | 10 | outdoor | adults | Burke et al., 2019 |
| Madagascar | Mahajanga | Mangatsa | -15.6667 | 46.6 | May 2003 | 1 | 36 | 14 | 0 | indoor | adults | Leong et al., 2003 |
| South_Africa | Tonga | Mangweni |  |  | August 1997 to May1998 | 1 | 2 | 0 | 2 | indoor/outdoor | adults | Govere et al., 2000 |
| South_Africa | Tonga | Martiens | -25.3728 | 31.80917 | August 1997 to May1998 | 223 | 20 | 0 | 115 | indoor/outdoor | adults | Govere et al., 2000 |
| Kenya | Malindi | Masheheni | -3.12791 | 40.10809 | June 1997 to May 1998 | 5 | 37 | 127 | 0 | indoor | adults | Mbogo et al., 2003 |
| Mozambique | Boane | Massingir | -23.8892 | 32.15056 | 14/02/2002 | 5 | 24 | 0 | 2 | indoor | adult | Cuamba et al., 2009 |
| Tanzania | Bagamoyo | Matimbwa | -6.5 | 38.867 | June to SSeptember 1996 | 4 | 15 | 217 | 0 | indoor | adult | Temu et al., 1998 |
| Mozambique | Boane | Mazoe | -16.2728 | 33.54556 | 13/07/2001 | 3 | 15 | 0 | 0 | indoor | adult | Cuamba et al., 2009 |
| South_Africa | Ehlanzeni | Mbombela | -25.4658 | 30.98528 | 2005 to 2015 | 1320 | 0 | 0 | 294 |  | adult/larvae | Mbokazi et al., 2018 |
| Kenya | Malindi | Mjanaheri | -3.08333 | 40.13333 | June 1997 to May 1998 | 3 | 18 | 22 | 0 | indoor | adults | Mbogo et al., 2003 |
| Mozambique | Boane | Mossuril | -14.9617 | 40.65889 | 01/08/2000 | 10 | 0 | 12 | 0 | indoor | adult | Cuamba et al., 2009 |
| South_Africa | Ehlanzeni | Nkomazi | -25.6667 | 31.66666 | 2005 to 2014 | 7898 | 539 | 0 | 2780 |  | adult/larvae | Mbokazi et al., 2018 |
| South_Africa | Tonga | Oompies |  |  | August 1997 to May 1998 | 4 | 1 | 0 | 2 | indoor/outdoor | adults | Govere et al., 2000 |
| Tanzania | Pemba | Pemba | -12.974 | 40.51775 | September 1959 to September 1960 | 5963 | 0 | 0 | 0 | outdoor | adults | Iyengar et al., 1962 |
| Mauritius | Pointe aux Piments | Pointe aux Piments | -20.0466 | 57.53771 | 1963 | 18 | 0 | 27 | 0 | outdoor | adults | Patterson et al., 1963 |
| Mozambique | Boane | Salamanga | -26.4725 | 32.61639 | 01/05/2000 | 59 | 22 | 0 | 0 | indoor | adult | Cuamba et al., 2009 |
| Mozambique | Boane | Salamanga | -26.4725 | 32.61639 | 12/05/2000 | 98 | 17 | 0 | 0 | outdoor | adult | Cuamba et al., 2009 |
| Tanzania | Handeni | Segera | -5.3 | 38.55 | April 1982 to January 1985 | 48 | 54 | 391 | 0 | indoor | adults | Mnzava et al., 1986 |
| Tanzania | Tanga | Tawalani | -4.86667 | 39.15 | April 1982 to January 1985 | 34 | 14 | 148 | 0 | indoor | adults | Mnzava et al., 1986 |
| Kenya | Kwale | Tsuini | -4.60108 | 39.16688 | June 1997 to May 1998 | 3 | 11 | 30 | 0 | indoor | adults | Mbogo et al., 2003 |
| South_Africa | Ehlanzeni | Umjindi | -25.746 | 31.034 | 2005 to 2016 | 360 | 0 | 0 | 171 |  | adult/larvae | Mbokazi et al., 2018 |
| Tanzania | Tanga | Vyeru | -4.95 | 39.133 | 1977 | 89 | 23 | 209 | 0 |  | adult | Bushrod et al., 1981 |
| Tanzania | Zanzibar | Zanzibar | -6.16667 | 39.23333 | April 1982 to January 1985 | 32 | 125 | 554 | 0 | indoor | adults | Mnzava et al., 1986 |
| Tanzania | Rufiji | Rufiji | -8 | 38.66667 | October 2003 to September 2004 | 39 | 135 | 388 | 0 | outdoor | adults | Kigadye et al., 2010 |
| South_Africa | NKunduse | NKunduse | -28.0833 | 32.33333 | January to May 1981 |  |  |  |  | outdoor | adults | Sharp et al., 1983 |
| South_Africa | Zululand | Makanis drift | -27 | 32.25 | 1983 |  |  |  |  |  | adults | Coetzee & Cross et al., 1983 |
| South_Africa | Gazankulu | Soutini | -23.4333 | 30.9 | 1983 |  |  |  |  |  | adults | Coetzee & Cross et al., 1983 |

**Abbreviations**

1. An me: *Anopheles merus*
2. An ar: *Anopheles arabiensis*
3. An ga: *Anopheles gambiae*
4. An qu: *Anopheles quadrinalatus*
